# A Review of Trikafta: Triple Cystic Fibrosis Transmembrane Conductance Regulator (CFTR) Modulator Therapy

**DOI:** 10.7759/cureus.16144

**Published:** 2021-07-03

**Authors:** Anas Zaher, Jude ElSaygh, Dalal Elsori, Hassan ElSaygh, Abdulsabar Sanni

**Affiliations:** 1 Internal Medicine, University of Debrecen, Debrecen, HUN; 2 Pediatrics, Rhode Island Hospital, Brown University, Rhode Island, USA; 3 Internal Medicine, Hennepin Healthcare, Minnesota, USA

**Keywords:** tezacaftor, cystic fibrosis triple therapy, cystic fibrosis therapy, cystic fibrosis, orkambi, lumacaftor, trikafta, elexacaftor, ivacaftor

## Abstract

Cystic fibrosis (CF) is a potentially fatal genetic disease that causes serious lung damage. With time, researchers have a more complete understanding of the molecular-biological defects that underlie CF. This knowledge is leading to alternative approaches regarding the treatment of this condition. Trikafta is the third FDA-approved drug that targets the F508del mutation of the CFTR gene. The drug is a combination of three individual drugs which are elexacaftor (ELX), tezacaftor (TEZ), and ivacaftor (IVA). This trio increases the activity of the cystic fibrosis transmembrane conductance regulator (CFTR) protein and reduces the mortality and morbidity rates in CF patients. The effectiveness of Trikafta, seen in clinical trials, outperforms currently available therapies in terms of lung function, quality of life, sweat chloride reduction, and pulmonary exacerbation reduction. The safety and efficacy of CFTR modulators in children with CF have also been studied. Continued evaluation of patient data is needed to confirm its long-term safety and efficacy. In this study, we will focus on reviewing data from clinical trials regarding the benefits of CFTR modulator therapy. We address the impact of Trikafta on lung function, pulmonary exacerbations, and quality of life. Adverse events of the different CFTR modulators are discussed.

## Introduction and background

Cystic fibrosis (CF) is a chronic, progressive, autosomal recessive disease that affects approximately 35,000 people in the United States [1,2]. The primary defect is a mutant CFTR gene causing a decrease or absence of cystic fibrosis transmembrane conductance regulator (CFTR) activity. CFTR protein is an epithelial anion transporter of chloride and bicarbonate. It regulates salt and water balance on the surface of cells and is encoded by the CFTR gene. A defect in the CFTR protein will cause pulmonary, gastrointestinal, pancreatic, and reproductive system diseases [3].

In CF, the CFTR protein is often expressed in epithelial cells' apical membranes. In CF patients, the secreted fluids are mostly mucus- and protein-rich [4]. CFTR proteins are expressed in the airway epithelia, para-nasal sinuses, pancreas, gut epithelia, biliary tree epithelia, vas deferens epithelia, and sweat duct epithelia [5]. However, the airway epithelia carry the highest levels of CFTR expression.

More than 1500 mutations in the CFTR gene have been discovered in the northern European and North American populations. Different mutations are divided into the 6 classes illustrated in Table 1. Class II mutations account for around two-thirds of the mutations found in CF patients. Usually, it’s due to the deletion of phenylalanine at the location 508 allele [2,6]. Protein misfolding and retention at the endoplasmic reticulum (ER) by the ER quality control system also affect the CFTR protein flow. Premature degradation occurs after such retention, preventing the protein from reaching the cell surface and severely reducing CFTR function [7].

**Table 1:** Classes of cystic fibrosis transmembrane conductance regulator (CFTR) gene mutations

Type of mutation	Type of CFTR mutation	Percent of people with CF who have at least 1 mutations.
Normal	CFTR protein is created and moves to the cell surface, allowing the transfer of chloride and water.	
Class I	No functional CFTR protein is created	22 percent
Class II	CFTR protein is created but misfolds, keeping it from moving to the cell surface. This is called a trafficking defect.	88 percent
Class III	CFTR protein is created and moves to the cell surface but the channel gate does not open. This is called a defective channel regulation.	6 percent
Class IV	CFTR protein is created and moves to the cell surface but the channel function is faulty. This is called decreased channel conductance.	6 percent
Class V	Normal CFTR protein is created and moves correctly to the cell surface but not enough amount of the protein. This is called reduced synthesis of CFTR.	5 percent
Class VI	CFTR protein is created but it does not work properly at the cell membrane. This is called decreased CFTR stability.	5 percent

In the absence of a functioning CFTR, cyclic adenosine monophosphate (cAMP)-dependent chloride and bicarbonate secretion into airway secretions will be impaired. Mucins become tethered to the bronchial apical surfaces and create an easy nidus for bacteria to invade [4]. Progressive lung failure due to opportunistic pathogen infection and mucosal chronic inflammation is a major source of morbidity and mortality in CF patients. CFTR protein deficiency also causes progressive fat and vitamin malabsorption and the inability to thrive [4].

As mentioned above, the major cause of morbidity and mortality in CF is respiratory failure [8]. Most patients ultimately develop progressive lung disease with airway mucus obstruction, bacterial infection, and inflammation despite intensive symptomatic therapies. Symptomatic therapies do not address the disease's molecular cause, often causing the disease to progress and complications to arise. Treatments that target the CFTR molecular defect are needed to halt the chain of events that leads to progressive lung disease [7]. It is important to note that new evidence surfaced suggesting that the CFTR protein plays an important role in many major respiratory disorders, such as asthma and chronic obstructive pulmonary disease [7].

## Review

A search was performed on databases including PubMed, Google scholar, and Journal of the European Cystic Fibrosis Society using keywords such as CF, ivacaftor (IVA), lumacaftor (LUM), Orkambi, elexacaftor (ELX), tezacaftor (TEZ), CF triple therapy, CF therapy. Articles published between 2014 - 2021 were reviewed.

In CF, the lungs are characterized by chronic inflammation that is driven by the infiltration of immune cells into the airways. The first cells to migrate into the airways are neutrophils. Although neutrophils are recruited to fight bacterial and fungal infections, their activation has the potential to damage surrounding lung tissue by releasing oxidants and protease enzymes [6]. In both the pediatric and adult populations, the overall cascade results in recurrent lung infections and pulmonary exacerbations which lead to weakened and impaired lungs [9].

After the discovery of the CF gene in 1989, researchers could figure out how different CFTR mutations cause biochemical and functional abnormalities in the CFTR protein. As a result, research into CFTR dysfunction and CF pathophysiology provided the knowledge needed for the production of pharmacologic compounds that target these various abnormalities. The discovery of the first clinical CFTR modulators was facilitated by the screening of large compound libraries in cell lines expressing different CFTR mutations. Representatives of two classes (potentiators and correctors) of CFTR-directed compounds have become available to treat patients with CF [10].

Based on their molecular mechanism of action, potentiators and correctors are the two types of FDA-approved CFTR modulators currently available. Potentiators bind to the CFTR protein in the plasma membrane, increasing the CFTR channel's opening frequency and ion conductance. IVA is the only approved CFTR potentiator, and they originally approved it for patients with the G551D mutation. CFTR correctors target the protein-folding defect that results from F508 gene deletion. Until recently, the only clinically approved corrector was LUM [11].

IVA a CFTR potentiator that enhances the gating frequency of CFTR channels on the cell surface. It was approved to improve the function of mutant CFTR protein. IVA's primary target is a CFTR protein whose glycine at position 551 has been replaced by aspartic acid (G551D). It is recommended to treat patients with multiple CFTR gating mutations (including G551D and other less common non-G551D class III mutations) [12].

The efficacy and safety of IVA were evaluated in two randomized placebo-controlled trials. Treatment with IVA resulted in a substantial improvement in FEV1 at 24 weeks in both trials, and these improvements persisted at 48 weeks. One trial showed that the mean improvement in percent expected FEV1 from baseline to 24 weeks was 10.6 percentage points higher in the IVA group compared to the placebo group. The other trial showed a 12.5 percentage points higher expected FEV1 in the IVA group compared to the placebo group. Patients who received the drug saw substantial changes in their CF symptoms and were 55% less likely to experience a pulmonary exacerbation than patients who received a placebo [13]. IVA reduced sweat chloride levels significantly but did not enhance lung function, meaning that a potentiator alone is not enough to save this mutated protein [14]. Some studies have shown that IVA increased innate immune cell activities, including the killing of Pseudomonas aeruginosa by neutrophils and monocytes [8].

In 2015, FDA approved the use of Orkambi in patients (aged 12 years or more) who are homozygous for F508del. Orkambi is a fixed-dose tablet containing a combination of LUM and IVA [11]. Two studies (TRAFFIC) and (TRANSPORT) researched the efficacy of Orkambi, and it showed an improvement in the percent predicted of FEV1 (ppFEV1). LUM-IVA increased ppFEV1 by 2.5%-2.6% (lower bound) to 4.0%-4.1% (upper bound) depending on the dose used [8,15] This combination, however, does not completely restore CFTR protein function and is ineffective in patients with the Phe508del-minimal function (MF) mutation [16]. Many experts believe that starting CFTR modulator therapy would alter the path of their CF lung disease. Indeed, clinical trials are gradually expanding the use of previously approved drugs to younger age ranges, in the hope that early CFTR modulation can delay or even preclude the development of pulmonary and extrapulmonary complications [17].

TEZ, the newest CFTR modulator, is a CFTR corrector that was recently approved by the FDA for use in conjunction with IVA. TEZ-IVA combination was found to be efficient in F508del heterozygous patients with a CFTR residual function mutation of the second allele. It enhances F508del-CFTR processing and trafficking. It also increases chloride transport in human bronchial epithelial cells from 2.5% to 8.1% of normal levels. Recent clinical trials have shown that the formulation of TEZ and IVA is marginally superior to its precursor, LUM-IVA, in terms of its improvements to FEV1. The adverse effect profile (including transient bronchoconstriction) and drug-drug interactions are also superior in the TEZ-IVA combination [10,14]

ELX is classified as a next-generation CFTR corrector because it differs from first-generation correctors like TEZ in terms of structure and mechanism. ELX was developed to address the need for an effective CF therapy for patients F508del-CFTR. Patients with genes that do not produce protein or produce proteins that are resistant to IVA or TEZ benefit most from ELX. ELX acts as a CFTR corrector by increasing the amount of mature CFTR proteins on the cell surface. Drugs like ELX help to improve a range of multi-organ CF symptoms, including lung function, nutritional status, and overall quality of life, when used in conjunction with CFTR potentiators, which improve the function of cell-surface CFTR proteins [18].

**Figure 1:**
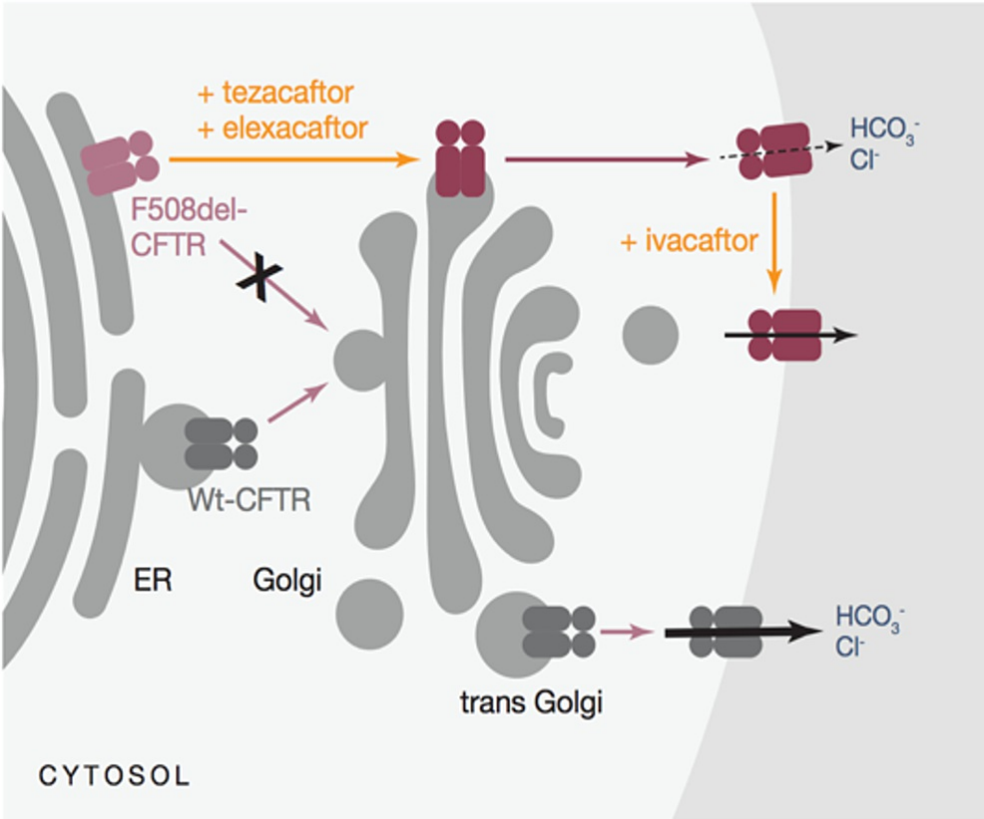
Mechanism of action of Trikafta on a cellular level [19]

In 2019, the FDA approved the use of Trikafta in patients with one copy of F508del-CFTR. Trikafta is the combination of two correctors (TEZ and ELX) and a potentiator (IVA). IVA directly targets mutant CFTR channel-forming protein. TEZ and ELX are thought to also work through direct interaction with mutant F508del-CFTR polypeptide, although evidence is lacking. The mechanism of action of Trikafta is illustrated in Figure 1. They target epithelial cells lining all of the tubular organs affected in CF, including the lungs, gastrointestinal tract, and pancreas. Trikafta costs $311,503 per year, and plans are likely to differ in terms of coverage among insurance companies [19].

FDA approval of Trikafta came after the results of two randomized, double-blind phase three studies (Trial 1-NCT03525444 and Trial 2-NCT03525548) conducted in CF patients aged 12 years and above with at least one F508del mutation [20]. The first trial was a 24-week placebo-controlled trial that enrolled around 400 patients. The second trial was a four-week active-controlled trial that enrolled around 100 patients with two similar F508del mutations. The trials looked at the pharmacokinetics, safety, and tolerability of ELX/TEZ/IVA over a two-week span. They also assessed the safety, tolerability, efficacy, and pharmacokinetics of the drug over a 24-week treatment period [21]. The studies showed a significant improvement in lung function, and respiratory-related quality of life, and a decrease in pulmonary exacerbations and sweat chloride after 24 weeks [21]. Details of the trials mentioned above will be discussed in the next section.

ELX-TEZ-IVA is supplied as a fixed-dose combination tablet of ELX 100 mg, TEZ 50 mg, and IVA 75 mg co-packaged with IVA 150-mg tablets. Adults and children over the age of 12 should administer two fixed-dose combination tablets each morning with a fat-containing meal. The evening dose should be separated by approximately 12 hours from morning administration and consists of one IVA 150-mg tablet taken with a fat-containing meal or snack. This medication is recommended to be taken with foods containing fat. Pancreatic enzymes can also be used for those who are pancreatic insufficient, to maximize efficacy [22].

1) A study of VX-445 combination therapy in CF subjects homozygous for the F508del (F/F)

This study evaluated the efficacy of VX-445 (ELX) in triple combination (TC) with TEZ and IVA in subjects with CF who are homozygous for the F508del mutation (F/F). The study enrolled 113 participants, of which six participants were included in the run-in period but were not dosed in the TC treatment period. Results were presented for 107 participants dosed in the TC treatment period. The duration of the trial was four months. It was a randomized, double-blind, controlled study. The inclusion criteria included: 12 Years and older CF patients, Homozygous for the F508del mutation (F/F) with Forced expiratory volume in 1 second (FEV1) value ≥40% and ≤90% of predicted mean for age, sex, and height. The two arms of the study were 52 patients treated with TEZ/IVA and 55 patients treated with VX-445/ TEZ/IVA. The primary outcome measured was the absolute change in ppFEV1.

The results showed that the change in ppFEV1 from baseline to four weeks was 10.4 percentage points for the triple therapy group and 0.4 for the TEZ/IVA arm (p<0.0001). Among the secondary outcomes measured, the absolute change in Sweat Chloride (SwCl) at week 4 was -43.4 in the treatment group vs 1.7 in the placebo group (p<0.0001). The absolute change in Cystic Fibrosis Questionnaire-Revised (CFQ-R) Respiratory Domain Score From baseline at week 4 was 16 units in those receiving triple therapy and -1.4 units in the placebo group (p<0.0001).

In terms of adverse events, no death was noted in both groups. Among the serious adverse events, one out of 55 patients from the triple therapy arm was diagnosed with infective pulmonary exacerbation of CF while one out of 52 patients experienced the same adverse event in the placebo group. Both groups reported diarrhea, abdominal pain, nausea, headache, cough, and fatigue [23].

2) A phase 3 study of VX-445 combination therapy in subjects with CF heterozygous for the F508del mutation and a minimal function mutation (F/MF)

This study evaluated the efficacy of VX-445 in triple combination with TEZ and IVA in subjects with CF who are heterozygous for F508del and a minimal function mutation (F/MF subjects). A total of 405 participants were enrolled in the study, of which two participants were enrolled but were not dosed in the triple combination treatment period. Results are presented for 403 participants dosed in the TC treatment period. The duration of the trial was 10 months. It was a randomized, double-blind, controlled trial. Inclusion criteria included: 12 Years and older CF patients, Heterozygous for the F508del mutation (F/MF) with FEV1 value ≥40% and ≤90% of predicted mean for age, sex, and height. The two arms were the placebo group and the treatment group receiving VX-445/TEZ/IVA. The primary outcome measured was the absolute change in ppFEV1.

The results showed a change in ppFEV1 from baseline at Week 4 was 13.6 percentage points for the treatment group and -0.2 for the placebo group (p<0.0001). Among the secondary outcomes measured, the absolute change in ppFEV1 from baselines at week 24 was 13.9 percentage points for the treatment group and -0.4 for the placebo group (p<0.0001).

The number of pulmonary exacerbations (PEx) from baseline at week 24 was 41 for the treatment group and 113 for the placebo group (p<0.0001). The absolute change in sweat chloride from baseline at Week 24 was -42.2 in the treatment group and -0.4 in the placebo group (p<0.0001). The absolute change in the CFQ-R respiratory domain score from Baseline at Week 24 was 17.5 points for the treatment group and -2.7 for the placebo group (p<0.0001). The absolute change in body mass index (BMI) from Baseline at Week 24 was 1.13 in the treatment group and 0.009 in the placebo group.

In terms of AE, no significant differences were found between groups in terms of adverse effects. No death was reported. 13.8 % of patients in the VX-445/TEZ/IVA group experienced serious side effects (intestinal obstruction, respiratory tract infections, rash, etc.) vs 20.9% in the placebo group. Common noted not-serious adverse effects include diarrhea, abdominal pain, vomiting, and fatigue [24].

3) Evaluation of VX 445/TEZ/IVA in CF subjects 6 through 11 years in age

This study evaluated the pharmacokinetics, safety, tolerability, efficacy, and pharmacodynamic effect of VX-445, TEZ, and IVA when dosed in TC in CF subjects 6 through 11 years of age. The study included 66 participants. Inclusion criteria were: CF patients aged 6 to 11 years, homozygous or heterozygous for F508del mutation, and FEV1 value ≥40% of predicted mean for age, sex, and height. The study was completed on August 7, 2020. However, the results are yet to be published [25].

Discussion

The discovery of the CFTR gene in 1989 paved the way for researchers to learn more about the structure, processing, and role of CFTR in health, revealing how multiple mutations in this epithelial anion channel cause a multiorgan disease. The approval of ivacaftor as the first CFTR modulator drug was a significant step forward and an important proof-of-concept for causal pharmacotherapy for this life-shortening genetic disorder on a larger scale. This CFTR potentiator restored around 50% of the CFTR function and showed improvement in lung function. And after the approval of the first corrector, lumacaftor combination therapy was used, and it was superior to monotherapy. Recent early phase clinical trials show that using triple-combination therapies with a second corrector compound is necessary to repair multiple defects in F508del processing. CF newborn screening has provided an opportunity to take advantage of the modulators in infants and young children, which have the potential to postpone or even avoid irreversible structural lung damage. However, CFTR modulator therapies may not help approximately 10% of the CF population.

The CFTR modulators IVA, LUM/IVA, TEZ/IVA, and ELX/TEZ/IVA have been shown to be effective not just in people with mild to severe CF, but even in people with advanced pulmonary disease, such as lung transplant applicants. Randomized controlled trials and open-label studies, which enrolled participants with more severe pulmonary disease than those inadvertently included in the randomized controlled trials, strongly showed this beneficial effect. It is widely accepted that the most significant effect of CFTR modulator therapy can be seen when it is initiated as early as possible in the disease's course when permanent lung damage is at the least serious. With the latest approval of the triple combination treatment for most people with CF by the FDA, we should expect more people with serious CF pulmonary disease to benefit from CFTR modulation.

## Conclusions

Trikafta is expected to cut the number of prescriptions prescribed every day in half and reduce the time spent on therapy per day. This will then cut down on regular care time, total cost, and treatment stress for people dealing with CF. Perhaps future experiments will prove this theory. Trikafta was revolutionary in improving the day-to-day activities of CF patients. A member of a support group on cysticfibrosis.com summarized her life-changing experience. She tried the Trikafta treatment for 12 months. She divided the treatment into two parts. Part 1 was the first six months. She referred to the treatment as a "silver bullet.” She noticed so many changes in her body and in her daily life because of Trikafta, ranging from deeper breaths to having more time and energy to lie on the floor and play blocks with her daughter. However, her major change was that she could talk on the phone while walking without becoming short of breath. In addition, she reported that during part 1, she had only one mild pulmonary exacerbation and it did not require hospital admission. Her PFT was increased by 10% after three months of treatment. Her cough vanished. Shortly after starting Trikafta, her 7% sodium chloride nebulizer treatment felt too strong. Her doctor diluted the treatment from 7% to 3.5% solution because the lungs were becoming too clear to have such a strong treatment. In part 2, she reported skin and nail changes, decreased joint and body aches, increased energy, and an increase in her appetite without nausea. This drug was truly magical in improving her lifestyle.

Additional studies for greater intervention groups and longer intervention times may be conducted to better show the treatment's potential in curing the underlying protein deficiency. For example, the clinical trial with CF patients less than 12 years old has been completed recently and results will be published soon. This treatment can avoid multi-organ manifestations of the disease and could enable young children with CF to live a normal life span. Implementing Trikafta is expected to lead to significant changes in the lives of people with CF. This level of CFTR modulation in such a large proportion of CF patients could have a significant impact on CF treatment.
